# The analysis of risk factors for fear and aggression during global crisis – a study based on Polish students among the Covid-19 pandemic.

**DOI:** 10.1192/j.eurpsy.2023.377

**Published:** 2023-07-19

**Authors:** A. Kawalec, K. M. Wilczyński, M. Janas-Kozik

**Affiliations:** ^1^Department of Psychiatry and Psychotherapy of Developmental Age, Medical University of Silesia, Katowice; ^2^Pediatric Centre of John Paul II Sosnowiec Sp. z o.o., Sosnowiec, Poland

## Abstract

**Introduction:**

Although the Covid-19 pandemic ceased in the numbers of the affected patients, especially the ones with severe manifestation of the disease, its influence on health still remains, affecting not only the somatic but also mental wellbeing. This global crisis impacted almost every person, but not equitable – the mental distress consisted of many, often synergic, risk factors which are not easily identifiable. The analysis of causal elements for aggression and fear deriving from the pandemic was conducted among Polish students, enabling thorough examination.

**Objectives:**

The aim of the study was to analyse the risk factors contributing to the deterioration of mental health, especially presented as elevated fear and aggression levels.

**Methods:**

Examination of fear and aggression levels was conducted on the group of Polish students using Fear of Covid and STAXI-2 questionnaires. Initially, 906 participants took part in the first round of the study. Four rounds were conducted, finally extracting a group of 231 participants tested in the four different time points of the pandemic in Poland, during the second and the third waves of the pandemic.

**Results:**

Among the studied factors that have impact on the decline in the state of the mental health, statistically significant were female sex, being overwhelmed by the amount of news found in various media – a phenomenon called “infodemic” - altogether with poor health condition, both of the participants’ and their relatives. Obtaining vaccination was a factor that lowered aggression in participants, but only the ones that were primarily eager to get one.

**Conclusions:**

The present deterioration of mental health in society was largely fuelled by the initial disturbance arising from global pandemic, general lockdown and financial crisis bound with it. The discrimination of risk factors for inefficient resilience was not possible on such a huge scale before. Unfortunately, as new challenges arise, the knowledge about the social groups most prone to mental crisis is necessary, as this is the only way to elaborate the proper procedures and guide a successful diagnostic process and treatment.

**Disclosure of Interest:**

None Declared

